# Privacy-Preserving Discovery of Topic-Based Events from Social Sensor Signals: An Experimental Study on Twitter

**DOI:** 10.1155/2014/204785

**Published:** 2014-04-03

**Authors:** Duc T. Nguyen, Jai E. Jung

**Affiliations:** Department of Computer Engineering, Yeungnam University, Gyeongsan 712-749, Republic of Korea

## Abstract

Social network services (e.g., Twitter and Facebook) can be regarded as social sensors which can capture a number of events in the society. Particularly, in terms of time and space, various smart devices have improved the accessibility to the social network services. In this paper, we present a social software platform to detect a number of meaningful events from information diffusion patterns on such social network services. The most important feature is to process the social sensor signal for understanding social events and to support users to share relevant information along the social links. The platform has been applied to fetch and cluster tweets from Twitter into relevant categories to reveal hot topics.

## 1. Introduction

Online services have been playing an important role in human life. Since these services help people to connect, transmit, share, and support diverse demands, most of people are using these services at work and daily activities. In particular, social network service (called SNS) has become more popular than the traditional communication media, for example, e-mail, SNS, video streams, and so on. The well-known SNSs are as follows (“How many people use the top social media Apps & Services” by Craig Smith, September 2013, on Digital Marketing Ramblings): Twitter (over 500 million users, more than 200 million active users), Facebook (1.15 billion users), Flickr (over 87 million users, 8 billion photos are shared), YouTube (over 1 billion users, 4 billion views per day), and so on.

These SNSs can be used with a number of types of social relationships, for example, a group of friends, a community of like-minded users, or business company and their customers. The users can share or announce their ideas, activities, and interesting events with others. Possible reasons are broad connection, diversity of applications, OS-independence, ease of use, and explosion in the number and kind of personal devices which are integrated wireless components, for example, smart phone, cameras, camcorders, and so on. It allows people to quickly update the latest information, their own opinion, or commentary toward a daily event. It opens a challenge issue of detecting which conversation topic trends are discussed and how to cluster incoming messages into relevant topic categories.

**Figure 1:**
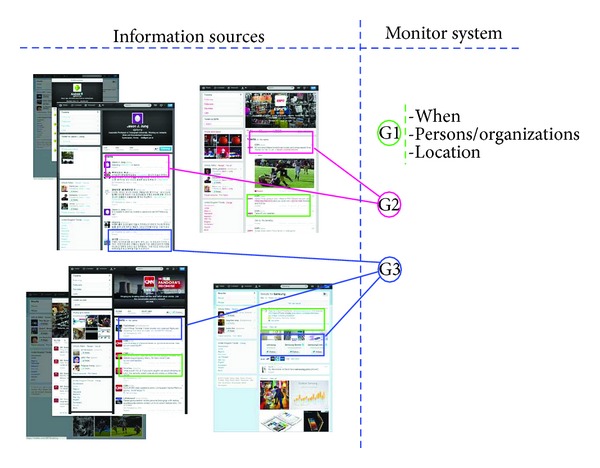
Example about demand to monitor and aggregate information.

For example, many news organizations use Twitter daily to post new short message with the purpose of sending information to a wide variety of people about hot news. A combination of information from multiple sources can show a completed image about one or a group of facts. Particularly, if it is interesting, more commentary and sharing action will be done by SNS's members. However, because of huge number of messages, it is difficult for people to follow all news and also to know what the significant information is. So, Figure 1 shows an example about a demand of tracking relevant message; we need an application that can notify what is discussed on SNSs right after these messages appeared on the data stream. And it is very useful if the application can extract embedded information in its content that will help to reduce time of monitoring and managing news on social networks.

With assumption that a social topic or an event will attract more attention from people when and around its first time of occurrence either in public or among a group of related users such as hobby group, technology community, and business network. Its signal can be expressed at the rapid increase in a short time range of

1. the number of messages described about a fact even if it is the personal opinions;
2. the response transactions which includes replying, discussion, or sharing action on an original message;
3. the frequency occurring of terms including meaningful keywords, named entities, or phrases.

In this paper, we only discuss topics, which relate to a community or to public scale, gathering a large enough number of messages about their context. These messages are collected in runtime on a data pipeline from Stream API (application programming interface) of SNS sites; the number of incoming message at a certain time can be large so that it requires an effective method to be processed, especially, when we use the rapidly changing symptoms of occurring frequency terms and the cooccurring frequency between them to look for topic trend candidates.

The outline of this paper is organized as follows. In Section 1, we introduce the problem and our approach of event detection and event-based clustering, in general; other related works are presented in Section 2; Section 3 shows an approach to build a system for monitoring and detecting topic trend; at the end, we show our experimental result in Section 4 and draw the conclusion and the future work in Section 5.

## 2. Related Work

In this work we deal with the problem of topic trend detection. Also, many researchers have tried to deal with the same problem in previous work [1–9]. Just like our social networks in real world, online social network is a complex organization that reflects participants' relationships in an open and almost unlimited environment to connect people around the world [10–12]. The number of posting messages on SNS is increasing day-by-day with the growth of computer, web application, and personal smart devices. It raises a challenge of analyzing and determining real-time and large-scale data for many studies from technical works to business applications [13, 14]. Most related researches to this work are about the problem of topic trend detection and clustering in social network where incoming messages are treated as a data stream [1, 2, 4, 8, 10, 15]; some works consider the incoming messages as the time series to track event by looking for changing data points [5, 6, 16].

## 3. Topic Detection and Clustering

We consider that SNS is a complex structure where people connect each each other via their relationships (e.g., friendship). Along these social structures, the information is sequentially transmitted from one to the other. This transmission sequence can be regarded as an instance of time series, where each information package is a data point with fully information for the next analysis phases.

### 3.1. Data Crawler

**Figure 2:**
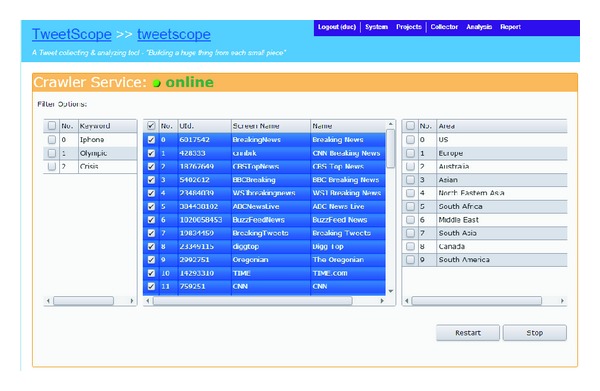
Example about demand to monitor and aggregate information.

The data stream is temporal; it contains some messages which are fully embedded with rich information about facts or topic trends. We assume that whenever a topic attracts attention of people, a lot of relevant messages will be posted in a short time range around its appearance time. Overtime, a topic trend will appear and disappear depending on its novelty and attractive content, so we call topics* life cycle* an approximate time range from a timestamp when people start to discuss something to the time when people do not talk about it so much. It needs an application which can work as an online application to fetch and classify incoming message as quick as possible, so that data service and web-based techniques are suitable to implement it.  Figure 2 is an illustration of the application GUI to manage the crawling functions, where data sources are selected from Twitter accounts of famous breaking news agent (http://twitaholic.com-Tool of tracking the most popular users of a certain microblogging/social network).

We discretize the data stream by using a sampling function with an interval Δ*t*; besides that depending on the performance of application system each sampling only collected a number *N* of messages which the system can process instantly. If messages come so quickly, they can be stored in a cache for next processes. The number of term distributed in a partition will be tracked as a series by time, where time concept is the partition index; the sequence is used to determine another relevant feature of a term or term pairs.

### 3.2. Topic Trend Detection

**Figure 3:**
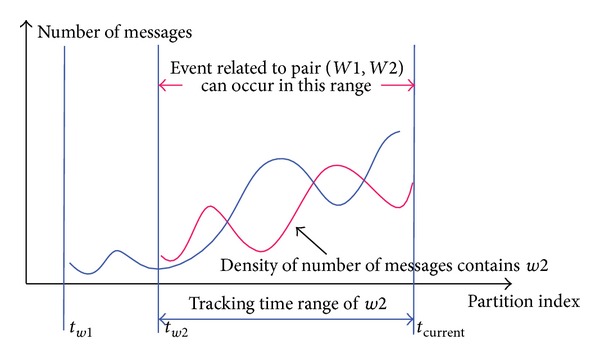
Terms tracking time range.

Around occurrence timestamp of a new topic, related messages will be fed into the system quickly; we found that these tweets contain some similar keyword or phrase which has strong meaning to a topic's content. Some SNSs restrict the length of posting message; thus people will try to use the most impressive term to describe facts; this is a one reason to make the appearance frequency of it be increased. The significant terms are surely to be used, if the message contains different factst. Besides that, no single keyword or phrase can describe at all a fact; it has to be combined together in a complete sentence; hence the cooccurring frequency of each individual term pair will also be increased.  Figure 3 shows an example about timestamp tracking of two terms *w*1 and *w*2, where *t*_*w*1_, *t*_*w*2_ is the first time, *w*1, *w*2 appear and are tracked, and the time range [*t*_*w*2_, *t*_current_] is the period where an potential topic trend related to *w*1 and *w*2 can occur. Using this feature, we propose a method to determine a potential topic trend by creating its characterization set, which is formed by collecting the most cooccurring term pairs, but ensure that each respective pair in the set has a high cooccurring frequency than a threshold. In other words, by detecting sudden changes in the sequence of number of term appearances, we can find when a new topic occurred.

## 4. Experimental Result

For evaluating the clustering method, we unify to specify fixed values: the time interval length of one stream partition Δ*t* is 30 minutes; the maximum number of data points in a partition *N* is not more than 100 items; not any topic trend candidates are tracked more than 2 days from the last appearance of its relevant message. However, we use two different input settings for our system depending on which kind of testing purpose and individual dataset is used. We found that our approach works well on an input dataset which contains a set of strong content-related tweets rather than a group of self-expressing tweets toward a fact. This is suitable for the main goal of this work to detect a trending topic which is drawing more attention from many people with a large enough number of interested news.

We have collected approximately 5 thousand tweets which are posted by Twitter accounts of breaking news agents such as CNN, BBC, and Reuters, from April 01, 2013, to July 31, 2013. The dataset is used to extract potential topic trends; then the result is evaluated by aligning extracted topics with well-known facts which occur around the time range. In Table 1, we show top 16 topic trends and their time range as an illustration for our experimental result.

## 5. Conclusion

In this paper, we have demonstrated our proposed system to detect topic trend from data stream of certain Twitter accounts. The applied method has advantage to detect new topic trend by clustering related message into corresponding categories using content-based methods and temporal information and propagating information of the messages among social community's members in runtime. The application is useful to help the user to track easily hot topics which are often discussed recently. But speed of performance still is problem; it needs to be improved by reimplementing better algorithms in future.

As a research limitation, we have realized that the communications on social media can be among heterogeneous communities (e.g., multilingual communities). Thus, as a future work, we want to consider the semantic identification methods [17] for the community of practice.

## Figures and Tables

**Table 1:** List of detected events from BND data set.

Number	Related events	Event date (GMT)		Topic terms
		Approximate date	Detected time	
1	Ricin letter is sent in US	April 18, 2013	April 18, 2013 00:41	Containing FBI, justice, letters, ricin, sent
2	fighters from PKK Kurdish separatist group leaving Turkey	April 25, 2013	April 25, 2013 13:15	fighters, Iraq, Kurdish, leaving, pkk, turkey
3	Boeing 787 Dreamliner arrives safely at Nairobi in the first commercial flight	April 27,2013	April 27,201307:55	The first, 787, Boeing, commercial, Dreamliner, flight
4	Bomb car attacks in Baghdad	May 27, 2013	May 27, 2013 15:11	Attacks, Baghdad, bombs, car, mainly, Shia
5	Protest in Turkey	May 31, 2013	May 31, 2013 15:05	Plans, square, Taksim
6	North Korea proposes talk with the USA	June 6, 2013	June 6, 2013 10:54	Korea, north, proposes, talks
7	Turkey using tear gas and water cannon again protesters	June 11, 2013	June 11, 2013 17:26	Cannon, enter, fired, gas, Gezi, Istanbul, park, protesters, tear, trying, water
8	Nelson Mandela's death	June 11, 2013	June 11, 2013 22:10	African, hospital, Mandela, Nelson, critical, improvement, stable
9	Lionel Messi and tax law infringement issue	June 12, 2013	June 12, 2013 13:14	4 m, 5 m, euros, father, filed, fraudulent, Lionel, Messi, returns, Spain, tax
10	Edward Snowden takes a flight to Moscow Airport	June 23, 2013	June 23, 2013 16:42	Asylum, Edward, Snowden, tweets
11	Snowden is in transit area of Moscow airport	June 25, 2013	June 25, 2013 14:49	Airport, confirms, fugitive, president, Snowden, transit
12	Asiana flight crashed	August 7, 2013	August 7, 2013 07:26	Asiana, flight, Francisco, landed, san
13	US military judge refuses to dismiss charges against Bradley Manning	August 18, 2013	August 18, 2013 14:20	Aiding, Bradley, charge, dismiss, enemy, giving, information
14	Catherine, the Duchess of Cambridge, and the royal baby boy	August 22, 2013	August 22, 2013 06:38	Baby, birth, boy, Cambridge, duchess, Kensington, palace
15	Crashed train in Spain	August 25, 2013	August 25, 2013 10:05	1944, 78, crash, deadliest, death, Galicia, rises, Spain, toll, train
16	Jewels are stolen in Cannes	August 28, 2013	August 28, 2013 12:01	136 m, 136 m, Cannes, double, estimate, French, jewels, stolen, Sunday, worth
